# Empirically derived dietary patterns and their association with mental health: a cross-sectional sample of Iranian migraine patients (2019–2020)

**DOI:** 10.1186/s40795-022-00522-x

**Published:** 2022-04-04

**Authors:** Arman Arab, Nahid Rafie, Amir Hadi, Fariborz Khorvash, Zahra Heidari, Gholamreza Askari

**Affiliations:** 1 Department of Community Nutrition, School of Nutrition and Food Science, Food Security Research Center, Isfahan University of Medical Sciences, Isfahan, Iran; 2 Department of Clinical Nutrition, School of Nutrition and Food Science, Food Security Research Center, Isfahan University of Medical Sciences, Isfahan, Iran; 3 Halal Research Center of IRI, FDA, Tehran, Iran; 4 Isfahan Neurosciences Research Center, Alzahra Hospital, Isfahan University of Medical Sciences, Isfahan, Iran; 5 Department of Biostatistics and Epidemiology, School of Health, Isfahan University of Medical Sciences, Isfahan, Iran

**Keywords:** Dietary pattern, Factor analysis, Depression, Anxiety, Stress

## Abstract

**Objectives:**

Based on a comprehensive search, we realized that there is no previous study conducted among migraine patients to assess the association between major dietary patterns and mental health. Therefore, the present study aims to explore the relationship between empirically-derived dietary patterns and depression, anxiety, and stress in a sample of Iranian migraine patients.

**Methods:**

A total of 262 migraine patients (20–50 years) were selected through simple random sampling method for this study. Dietary intake during the previous year was determined using a validated 168-item, semi-quantitative food frequency questionnaire (FFQ), and major dietary patterns were derived using principal component analysis (PCA). For mental health evaluation, authors used the Depression, Anxiety, and Stress Scales (DASS- 21) questionnaire. Statistical analysis included multinomial logistic regression analysis and results were expressed as odds ratio (OR) with a 95% confidence interval (CI).

**Results:**

We identified three major dietary patterns including “traditional”, “western”, and “healthy”. After controlling for potential confounders, those in the highest tertile of the healthy dietary pattern had lower odds of depression (OR = 0.44, 95% CI: 0.22, 0.88; P for trend: 0.030) and stress (OR = 0.50, 95% CI: 0.25, 0.99; P for trend: 0.049). However, no significant association was observed between western and traditional dietary patterns and mental disorders.

**Conclusions:**

In summary, significant associations were documented between healthy dietary patterns and risk of depression and stress. Current findings urge migraine patients to increase their intakes of fruits, vegetables, eggs, whole grains, nuts and seeds, meat, and poultry and reduce the intake of fast foods and snacks, processed meat, fish, cola drink, condiments, dairy, and vegetable pickles to diminish the chance of depression and stress.

## Introduction

Migraine is known as a multifactorial disorder, with hormonal, genetic, environmental, sleep, dietary, and psychological aspects playing different roles in each individual [1]. The social and individual impact of migraine is also remarkable and diverse, including different sorts of psychiatric impairments [2]. It has been reported that mood disorders are among the most important psychiatric conditions associated with migraine including its prevalence, prognosis, treatment, and clinical outcomes [3]. Previous datasets have indicated that migraineurs suffer from mood disorders two to ten folds more than the general population [4, 5]. Moreover, these disorders are associated with reduced quality of life and a higher rate of suicide among both migraineurs and the general population [6–9]. Therefore, it is imperative to carefully inspect the factors that could diminish mood disorders in migraine patients.

To improve this health condition, studies have shown the possible favorable effect of some nutrients such as folate, vitamins B6, B12, E, C, D, and omega-3 fatty acids on mental health [10]. However, in real life, people do not consume nutrients and foods separately but in the form of meals containing combinations of many nutrients and foods that possibly inter-correlate and interact with each other [11]. So, the term “dietary patterns” was introduced over the past decade which represents a full picture of interactions between food items and nutrients in the diet [12].

The literature reviewed shows that many studies have been conducted on the association of various dietary patterns [13, 14] or dietary scores (i.e., healthy eating index [15, 16], Mediterranean diet [17], DASH diet [18], etc.) with the risk of mental disorders, although these studies have been done on migraine-free individuals. In a study on British women, a ‘whole food’ dietary style reduced the risk of depression, while a ‘processed food’ dietary pattern was associated with greater risk [19]. Higher adherence to a ‘traditional’ dietary pattern (high load of whole grains, fruit, vegetables, fish, and meat) was also shown to be associated with a lower risk of depression among the Australian population. Similar reports are available from China [20], Norway [21], and Iran [22].

Based on a comprehensive search, we realized that the association between dietary patterns and mental health has not been investigated among patients with migraine, and also limited data are available on Middle Eastern populations. Therefore, the present cross-sectional study aims to explore the relationship between empirically-derived dietary patterns and mental health including depression, anxiety, and stress in a sample of Iranian migraine patients. We assume that healthy dietary patterns may have potential advantages for mental health while unhealthy types pose adverse effects to psychological health.

## Methods

### Study design and population

A cross-sectional sample of adult migraineurs residing in Isfahan, Iran, was recruited between August 2019 and June 2020. Potential patients were assessed consecutively and selected through a simple random sampling method from two neurology clinics associated with Isfahan University of Medical Sciences. Adult migraine patients (20–50 years) with a confirmed diagnosis by a neurologist (F.K) pursuant to the International Classification of Headache Disorders 3 (ICHD3) criteria; with body mass index (BMI) of 18.5 to 30 were included. Those with a diagnosis of hypertension, diabetes, cancer, thyroid disease, hepatic or renal conditions due to possible disease-related changes in diet were considered ineligible. Other exclusion criteria were reported to use of herbal and dietary supplements including coenzyme Q10, riboflavin, magnesium, or feverfew, and under-or over-reporting of calorie intake based on normal energy intake of 800–4200 kcal/day [23]. Participants signed informed written consent, and the study protocol received the favorable opinion of the research ethics committee of Isfahan University of Medical Sciences (IR.MUI.RESEARCH.REC.1398.352) and the study was also conducted according to the Declaration of Helsinki.

### Dietary assessment

Researchers used a valid 168-item, semi-quantitative food frequency questionnaire (FFQ) to determine the usual dietary intake of the participants during the previous year [24–26]. This questionnaire has a list of foods, along with a standard serving size for each. The participants were instructed to report their intake on the basis of daily, weekly, or monthly of a given serving size of each food item. By using household measures, portion sizes of consumed foods were converted to grams [27]. FFQ was completed through a face-to-face interview by a trained dietitian and data were analyzed by Nutritionist IV software (First Databank, Hearst Corp, San Bruno, CA, USA). To detect major dietary patterns, each food item was assigned to one of 21 predefined food groups (Table 1). These food groups were chosen based on the similarity of the nutrient profile and their relationship with psychological health [28].

**Table 1:** Food groups using for dietary pattern analysis and factor loadings for each of the identified dietary patterns^a^

Food groups	Food items	Dietary pattern		
		Healthy	Traditional	Western
Fruits	Different kinds of fresh fruits, dry fruits, natural fruit juice, fruit conserves, industrial juice	0.63	–	–
Vegetables	Yellow, green leafy, and all other types of vegetables	0.61	–	–
Egg	Eggs	0.60	–	–
Whole grains	Dark Iranian bread (e.g., barbari, sangak, taftun), cooked potato, barely	0.56	–	–
Nuts and seeds	Walnuts, peanuts, pistachios, hazelnuts, almonds, seeds	0.43	–	–
Meat	Beef, lamb and all type of organ meats	0.39	–	–
Poultry	Chicken	0.35	–	–
Solid oils	Hydrogenated vegetable oil, animal oil, margarine, butter, mayonnaise	–	0.60	–
Sweets	Confectionary products, sugar, jam, honey, candy	–	0.49	–
Caffeine	Tea, coffee, chocolate, cacao	–	0.47	–
Legumes	Different kinds of beans, peas, and lentil	–	0.46	–
Refined grains	White bread including lavash, baguette, rice, pasta, vermicelli, cake, and biscuits	–	0.41	–
Healthy oils	Vegetable oils, olive	–	−0.50	–
Fast foods and snacks	Pizza, potato chips, French fries, cheese snacks	–	–	0.76
Processed meat	Sausage, hamburger, other	–	–	0.72
Fish	All types of fish, canned tuna	–	–	0.53
Cola	All types of cola drinks	–	–	0.52
Condiment	Seasoning, salt, ketchup, lime juice	–	–	0.43
Dairy	All type of milk, yoghurt, cheese, ice cream, dough, curd	–	–	0.38
Vegetable pickles	All types of vegetable pickles	–	–	0.30

^a^Factor loadings of < 0.3 have been removed to simplify the table

### Mental health assessment

A previously validated questionnaire in the Iranian population [29], the Depression, Anxiety, and Stress Scale (DASS- 21), was used to assess mental health. Scores for each question range from 0 (did not apply to me at all) to 3 (applied to me very much or most of the time) and the sum of scores for each domain is multiplied by 2 to evaluate the original 42-item DASS [30]. Overall points for depression (0–9, 10–13, 14–20, 21–27, ≥28), anxiety (0–7, 8–9, 10–14, 15–19, ≥20) and stress (0–14, 15–18, 19–25, 26–33, ≥34) were categorized as normal, mild, moderate, severe, and extremely severe [31].

### Assessment of other variables

Researchers obtained further information about marital status, number of family members, gender, age, family history of migraine, time since diagnosis of migraine, type of migraine (episodic/chronic), and medication by a demographic questionnaire. Chronic migraine was defined as having migraine headaches ≥15 days of a month with at least 8 days with typical features of a migraine headache. The International Physical Activity Questionnaire (IPAQ), a self-administered, 7-day recall, previously validated instrument among the Iranian population [32], indicated the physical activity status and expressed it as metabolic equivalent hours per day (METs h/day). The weight and height of participants were measured according to the standard methods using a digital scale (Omron BF511 (Omron Corp, Kyoto, Japan)) and an upstretched tape, respectively, and BMI was calculated with a related equation [33].

### Statistical analysis

The sample size was estimated on the basis of similar studies and a formula for cross-sectional design using α = 0.05, β = 0.95, *r* = 0.25, and a drop-out rate of 10% was used that yielded 265 subjects [34]. We used exploratory factor analysis based on the principal component estimation method to identify major dietary patterns on the basis of 21 FFQ-derived food groups. In the first step, the quality of dietary data was assessed using the Kaiser-Meyer-Olkin criterion and the Bartlett’s test of sphericity test. The number of factors (dietary patterns) was determined using a scree plot and eigenvalues (> 1.5) in conjunction with the natural interpretation of the factors. Finally, factors were rotated using an orthogonal (varimax) rotation to minimize the correlation between the factors and improve interpretability. The derived dietary patterns (factors) were labeled on the basis of earlier literature and the loaded food groups in each factor. The score of each dietary pattern was calculated by summing the intake of food groups weighted by their factor loadings. The participants were categorized based on the tertiles of dietary pattern scores. The distribution of subjects across tertiles of dietary patterns in terms of categorical variables was examined using the Chi-square test. The differences of continuous variables across categories of dietary patterns were assessed by one-way ANOVA. So as to examine the relationship between dietary patterns and mental health, we used ordinal logistic regression analysis in different models. First, we adjusted for family size (continuous), marital status (single/married), gender, age (continuous), smoking (current smoker/non-current smoker), migraine headache index score (continuous), physical activity (continuous), and mean arterial pressure (continuous). Further adjustment was made for total energy intake (continuous) and BMI (continuous) in the last model. Data analyses were performed using SPSS version 21 (IBM Corp, Armonk, NY, USA). *P*-values < 0.05 were considered statistically significant.

## Results

A total of 262 out of 298 initial participants completed the study (response rate 87%). Overall, 262 patients diagnosed with migraine contributed to the current study. The mean age of the study population was 36.10 years with a BMI of 25.55 kg/m^2^. They consisted of 224 (85.5%) women; 212 (80.9%) were married, and 15 (5.7%) were current smokers.

Three major dietary patterns were as follows: (i) a ‘western’ dietary pattern rich in fast foods and snacks (0.76), processed meat (0.72), fish (0.53), cola drink (0.52), condiments (0.43), dairy (0.38), and vegetable pickles (0.30); (ii) a ‘traditional’ dietary pattern comprised mainly of solid oils (0.60), sweets (0.49), caffeine (0.47), legumes (0.46), and refined grains (0.41); and (iii) a ‘healthy’ dietary pattern characterized by high intake of fruits (0.63), vegetables (0.61), egg (0.60), whole grains (0.56), nuts and seeds (0.43), meat (0.39), and poultry (0.35) (Table 1). The extracted dietary patterns corresponded to 34% of the total variance in dietary intakes of the study population.

The general characteristics of the study participants and data regarding medication and dietary intakes across tertiles of dietary patterns are presented in Table 2. Those in the highest tertile of the healthy dietary pattern had higher physical activity, higher intake of tricyclic antidepressants, and consumed more calories, protein, fat, and carbohydrate, compared to those in the lowest tertile (all *P* values < 0.05). When compared to the lowest tertile of traditional dietary pattern, there were more men in the highest tertile, the family size was bigger, and they had a higher intake of calories, protein, fat, and carbohydrate (all *P* values < 0.05). Patients with the highest adherence to the western dietary pattern in relation to the lowest adherence were less likely to be female and married. Moreover, these subjects had lower age, higher height, and consumed higher amounts of energy, carbohydrate, fat, and protein (all *P* values < 0.05). No significant differences were observed in terms of other general characteristics across tertiles of the healthy, western, and traditional dietary patterns (all *P* values > 0.05).

**Table 2:** Characteristics of study population across tertiles (T) of major dietary patterns

	Healthy dietary pattern				Traditional dietary pattern				Western dietary pattern			
	T1	T2	T3	***P*** value	T1	T2	T3	***P*** value	T1	T2	T3	***P*** value
N	87	88	87		87	88	87		87	88	87	
Age (y)	36.54 ± 1.18	36.00 ± 1.01	36.01 ± 1.00	0.918	35.63 ± 1.11	36.19 ± 1.00	36.73 ± 1.08	0.765	40.34 ± 0.93	37.84 ± 0.94	30.36 ± 0.96	< 0.001
Female	72 (82.7)	69 (78.4)	64 (73.6)	0.075	75 (86.2)	73 (82.9)	57 (65.5)	< 0.001	74 (85.0)	68 (77.3)	63 (72.4)	0.014
Married	63 (72.4)	66 (75.0)	66 (75.8)	0.543	62 (71.3)	66 (75.0)	67 (77.0)	0.311	73 (83.9)	70 (79.5)	52 (59.8)	< 0.001
Current smoker	11 (12.6)	13 (14.8)	13 (14.9)	0.513	13 (14.9)	10 (11.4)	14 (16.1)	0.744	9 (10.3)	14 (15.9)	14 (16.1)	0.102
Number of family members	3.50 ± 0.11	3.38 ± 0.10	3.67 ± 0.12	0.199	3.30 ± 0.11	3.52 ± 0.10	3.73 ± 0.11	0.023	3.69 ± 0.11	3.41 ± 0.10	3.45 ± 0.11	0.152
Weight (kg)	65.94 ± 1.28	68.61 ± 1.25	68.94 ± 1.33	0.201	66.60 ± 1.22	66.56 ± 1.14	70.34 ± 1.46	0.060	67.84 ± 1.18	66.73 ± 1.22	68.92 ± 1.47	0.490
Height (cm)	161.39 ± 0.93	163.07 ± 0.91	164.19 ± 0.95	0.107	161.97 ± 0.86	162.32 ± 0.89	164.36 ± 1.04	0.151	160.76 ± 0.90	162.39 ± 0.90	165.50 ± 0.93	0.001
BMI (kg/m^2^)	25.30 ± 0.42	25.80 ± 0.41	25.52 ± 0.40	0.695	25.34 ± 0.39	25.31 ± 0.42	25.96 ± 0.41	0.454	26.24 ± 0.39	25.31 ± 0.42	25.07 ± 0.41	0.107
Physical activity (MET/h/d)	5.05 ± 1.23	9.98 ± 2.30	15.12 ± 3.62	0.024	7.52 ± 1.58	10.16 ± 1.92	12.47 ± 3.78	0.410	8.00 ± 2.09	12.27 ± 3.43	9.88 ± 2.09	0.514
MAP (mmHg)	88.61 ± 0.82	87.66 ± 0.90	88.37 ± 0.79	0.707	88.26 ± 0.82	86.81 ± 0.98	89.57 ± 0.64	0.065	88.47 ± 0.75	88.79 ± 0.79	87.38 ± 0.95	0.463
MHIS	39.34 ± 6.08	61.16 ± 9.37	55.42 ± 7.26	0.118	60.72 ± 7.95	48.94 ± 7.89	46.26 ± 7.35	0.374	56.12 ± 8.83	55.50 ± 7.01	44.31 ± 7.26	0.480
Medications												
Taking beta-blockers	37 (42.5)	36 (40.9)	34 (39.1)	0.611	35 (40.2)	36 (40.9)	36 (41.4)	0.865	33 (37.9)	37 (42.0)	37 (42.5)	0.498
Taking topitamate	10 (11.5)	11 (12.5)	14 (16.1)	0.162	15 (17.2)	10 (11.4)	10 (11.5)	0.081	13 (14.9)	11 (12.5)	11 (12.6)	0.485
Taking TCAs	33 (37.9)	43 (48.9)	45 (51.7)	0.046	37 (42.5)	44 (50.0)	40 (46.0)	0.619	39 (44.8)	42 (47.7)	40 (46.0)	0.868
Taking TeCAs	12 (13.8)	9 (10.2)	8 (9.2)	0.060	10 (11.5)	11 (12.5)	8 (9.2)	0.348	7 (8.0)	12 (13.6)	10 (11.5)	0.159
Taking SNRIs	10 (11.5)	13 (14.8)	10 (11.5)	> 0.99	10 (11.5)	13 (14.8)	10 (11.5)	> 0.99	9 (10.3)	13 (14.8)	11 (12.6)	0.450
Taking sodium valproate	18 (20.7)	19 (21.6)	14 (16.1)	0.330	18 (20.7)	14 (15.9)	19 (21.8)	0.808	17 (19.5)	17 (19.3)	17 (19.5)	> 0.99
Taking triptans	19 (21.8)	24 (27.3)	16 (18.4)	0.509	20 (23.0)	20 (22.7)	19 (21.8)	0.826	18 (20.7)	22 (25.0)	19 (21.8)	0.826
Taking gabapentin	20 (23.0)	17 (19.3)	14 (16.1)	0.144	20 (23.0)	18 (20.4)	13 (14.9)	0.088	17 (19.5)	18 (20.4)	16 (18.4)	0.808
Taking benzodiazepine	12 (13.8)	12 (13.6)	9 (10.3)	0.257	10 (11.5)	13 (14.8)	10 (11.5)	> 0.99	11 (12.6)	11 (12.5)	11 (12.6)	> 0.99
Macronutrient intake												
Total energy intake (kcal/d)	2380.93 ± 64.15	2564.83 ± 64.82	3015.63 ± 67.72	< 0.001	2430.56 ± 73.32	2640.99 ± 64.37	2889.84 ± 69.84	< 0.001	2271.05 ± 51.22	2670.14 ± 62.69	3020.20 ± 73.54	< 0.001
Carbohydrate (g/d)	320.08 ± 10.82	348.78 ± 10.70	415.92 ± 11.42	< 0.001	324.23 ± 11.82	363.45 ± 10.61	397.11 ± 11.83	< 0.001	303.03 ± 8.23	368.29 ± 11.40	413.47 ± 12.08	< 0.001
Protein (g/d)	57.71 ± 2.35	70.72 ± 2.52	91.76 ± 3.31	< 0.001	69.03 ± 3.29	74.70 ± 3.25	76.45 ± 3.04	0.232	57.37 ± 2.15	71.44 ± 2.64	91.38 ± 3.36	< 0.001
Fat (g/d)	103.54 ± 3.52	107.28 ± 2.84	122.10 ± 3.26	< 0.001	104.36 ± 2.92	109.06 ± 3.21	119.50 ± 3.63	0.004	99.98 ± 3.13	110.58 ± 3.08	122.37 ± 3.30	< 0.001

Data are presented as mean ± standard error or number (% within tertiles of dietary pattern scores)

*P*-value obtained from chi-square analysis for categorical variables and analysis of variance (ANOVA) for continuous variables

*BMI* Body mass index, *MAP* Mean Arterial Pressure, *MHIS* Migraine Headache Index Score, *TCA* Tricyclic Antidepressants, *TeCA* Tetracyclic Antidepressant, *SNRI* Serotonin-Norepinephrine Reuptake Inhibitor

*P* < 0.05 was considered statistically significant

General characteristics of the study population across categories of mental health are shown in Table 3. No significant differences were observed in terms of baseline variables throughout the categories of mental health (depression, anxiety, stress) (all *P* values > 0.05).

**Table 3:** Characteristics of study population across categories of mental health

	Depression				Anxiety				Stress			
	Normal	Moderate	Extremely severe	***P*** value	Normal	Moderate	Extremely severe	***P*** value	Normal	Moderate	Extremely severe	***P*** value
Age (y)	35.48 ± 0.93	37.04 ± 1.06	36.66 ± 1.23	0.777	35.36 ± 1.00	35.62 ± 1.48	35.66 ± 0.87	0.167	35.64 ± 1.08	34.59 ± 1.21	36.00 ± 1.34	0.535
Female	68 (81.9)	57 (86.4)	45 (90.0)	0.182	58 (84.1)	34 (79.1)	86 (90.5)	0.121	45 (84.9)	37 (84.1)	46 (88.5)	0.322
Married	65 (8.3)	57 (86.4)	42 (84.0)	0.555	55 (79.7)	32 (74.4)	79 (83.2)	0.272	42 (79.2)	35 (79.5)	42 (80.8)	0.659
Current smoker	5 (6.0)	3 (4.5)	1 (2.0)	0.185	2 (2.9)	5 (11.6)	5 (5.3)	0.835	2 (3.8)	3 (6.8)	2 (3.8)	0.823
Number of family members	3.31 ± 0.10	3.25 ± 0.11	3.46 ± 0.13	0.089	3.55 ± 0.11	3.34 ± 0.15	3.34 ± 0.10	0.680	3.54 ± 0.14	3.31 ± 0.14	3.21 ± 0.13	0.381
Weight (kg)	67.63 ± 1.09	68.95 ± 1.39	67.68 ± 1.39	0.881	66.11 ± 1.20	68.86 ± 1.89	68.48 ± 1.10	0.538	67.23 ± 1.37	67.63 ± 1.68	67.86 ± 1.46	0.987
Height (cm)	162.78 ± 0.82	163.22 ± 0.98	161.86 ± 0.96	0.792	161.89 ± 0.89	164.83 ± 1.37	163.03 ± 0.76	0.354	161.90 ± 1.05	163.22 ± 1.28	162.87 ± 1.12	0.884
BMI (kg/m^2^)	25.53 ± 0.37	25.82 ± 0.41	25.82 ± 0.47	0.583	25.20 ± 0.39	25.29 ± 0.56	25.75 ± 0.36	0.774	25.65 ± 0.45	25.40 ± 0.56	25.53 ± 0.43	0.990
Physical activity (MET/h/d)	8.22 ± 2.81	9.79 ± 2.49	8.80 ± 2.25	0.995	9.76 ± 3.38	8.59 ± 2.59	8.39 ± 1.54	0.973	9.82 ± 4.27	4.57 ± 1.46	9.63 ± 2.45	0.682
MAP (mmHg)	87.79 ± 0.77	87.54 ± 0.90	87.27 ± 1.22	0.775	88.80 ± 0.73	88.15 ± 1.16	86.38 ± 0.87	0.163	87.81 ± 0.90	88.04 ± 1.13	87.41 ± 1.21	0.867
MHIS	52.44 ± 8.02	46.33 ± 5.94	63.34 ± 11.19	0.619	43.28 ± 6.76	41.29 ± 7.49	67.83 ± 7.88	0.078	38.62 ± 5.65	62.10 ± 10.55	55.40 ± 9.72	0.343

Data are presented as mean ± standard error or number (% within categories of mental health)

*P*-value obtained from chi-square analysis for categorical variables and analysis of variance (ANOVA) for continuous variables

*BMI* Body mass index, *MAP* Mean Arterial Pressure, *MHIS* Migraine Headache Index Score

*P* < 0.05 was considered statistically significant

The distribution of the study population in terms of depression, anxiety, and stress across categories of different dietary pattern scores is provided in Table 4. There were no significant differences regarding the distribution of subjects across tertiles of healthy, western, and traditional dietary patterns in terms of any of the intended parameters of mental health (all *P* values > 0.05).

**Table 4:** Mental health of participants across tertiles (T) of major dietary patterns

	Healthy dietary pattern				Traditional dietary pattern				Western dietary pattern			
	T1	T2	T3	***P***-value	T1	T2	T3	***P***-value	T1	T2	T3	***P***-value
Depression				0.43				0.72				0.09
Normal	20 (23.0)	24 (27.3)	30 (34.5)		25 (28.9)	21 (23.9)	28 (32.2)		28 (32.2)	18 (20.4)	28 (32.2)	
Mild	16 (18.4)	13 (14.8)	15 (17.2)		15 (17.2)	14 (15.9)	15 (17.2)		14 (16.0)	18 (20.4)	12 (13.8)	
Moderate	24 (27.5)	25 (28.4)	18 (20.7)		19 (21.8)	29 (33.0)	19 (21.8)		28 (32.2)	19 (21.6)	20 (23.0)	
Severe	6 (6.9)	12 (13.6)	8 (9.2)		9 (10.3)	9 (10.2)	8 (9.2)		5 (5.8)	11 (12.5)	10 (11.5)	
Extremely severe	21 (24.2)	14 (15.9)	16 (18.4)		19 (21.8)	15 (17.0)	17 (19.6)		12 (13.8)	22 (25.0)	17 (19.6)	
Anxiety				0.54				0.26				0.95
Normal	26 (29.9)	18 (20.4)	23 (26.4)		25 (28.9)	20 (22.7)	22 (25.3)		24 (27.5)	21 (23.9)	22 (25.3)	
Mild	6 (6.9)	11 (12.5)	8 (9.2)		8 (9.2)	6 (6.8)	11 (12.6)		7 (8.1)	9 (10.2)	8 (9.2)	
Moderate	15 (17.2)	20 (22.7)	13 (15.0)		16 (18.4)	16 (18.2)	16 (18.4)		15 (17.2)	14 (15.9)	19 (21.8)	
Severe	10 (11.5)	11 (12.5)	15 (17.2)		11 (12.6)	19 (21.6)	7 (8.1)		13 (15.0)	15 (17.0)	10 (11.5)	
Extremely severe	30 (34.5)	28 (31.9)	28 (32.2)		27 (31.0)	27 (30.7)	31 (35.6)		28 (32.2)	29 (33.0)	28 (32.2)	
Stress				0.06				0.49				0.56
Normal	12 (13.8)	14 (15.9)	26 (29.9)		18 (20.7)	18 (20.4)	18 (20.7)		19 (21.8)	16 (18.2)	19 (21.8)	
Mild	15 (17.2)	22 (25.0)	10 (11.5)		19 (21.8)	13 (14.8)	15 (17.2)		21 (24.2)	12 (13.6)	13 (15.0)	
Moderate	17 (19.6)	16 (18.2)	12 (13.8)		9 (10.3)	17 (19.3)	18 (20.7)		14 (16.0)	15 (17.0)	16 (18.4)	
Severe	27 (31.0)	17 (19.3)	20 (23.0)		24 (27.5)	18 (20.4)	22 (25.3)		20 (23.0)	25 (28.4)	19 (21.8)	
Extremely severe	16 (18.4)	19 (21.6)	19 (21.8)		17 (19.6)	22 (25.0)	14 (16.0)		13 (15.0)	20 (22.7)	20 (23.0)	

Data are presented as number (% within tertiles of dietary pattern scores)

*P*-value obtained from chi-square analysis

*P* < 0.05 was considered statistically significant

The results of the Pearson correlation coefficient test for the association between mental health and dietary patterns are shown in Table 5. As can be seen, no significant correlation was detected between the dietary patterns (healthy, western, traditional) and mental health (depression, anxiety, stress).

**Table 5:** Correlation coefficient for the association between mental health (depression, anxiety and stress) and major dietary patterns

	Healthy dietary pattern	Traditional dietary pattern	Western dietary pattern
Depression score	−0.018 (*P* = 0.786)	−0.052 (*P* = 0.442)	0.040 (*P* = 0.550)
Anxiety score	0.070 (*P* = 0.301)	0.091 (*P* = 0.181)	0.042 (*P* = 0.539)
Stress score	−0.025 (*P* = 0.712)	0.063 (*P* = 0.351)	0.034 (*P* = 0.617)

Data are presented as correlation coefficient (*P*-value)s

*P* < 0.05 was considered statistically significant

*P*-value obtained from Spearman correlation test

The multivariable-adjusted odds ratio for depression, anxiety, and stress across tertiles of major dietary patterns are indicated in Table 6. In the crude model, individuals in the top tertile of healthy dietary patterns tended to have lower odds of depression (OR = 0.56, 95% CI: 0.31, 1.01; P_trend_ = 0.107). After controlling for sex, age, marital status, smoking, number of family members, migraine headache index score, mean arterial pressure, physical activity, BMI, and total energy intake, those in the highest tertile of the healthy dietary pattern had lower odds of depression (OR = 0.44, 95% CI: 0.22, 0.88; P_trend_ = 0.030). We found no significant association between healthy dietary patterns and risk of anxiety even after controlling for confounding factors (OR = 0.70, 95% CI: 0.35, 1.39; P_tend_ = 0.357). Adherence to the healthy dietary pattern was not associated with the risk of stress in the crude model (OR = 0.64, 95% CI: 0.35, 1.15; P_trend_ = 0.222); however, adjustment for age, marital status, sex, smoking, number of family members, migraine headache index score, mean arterial pressure, physical activity, BMI, and total energy intake made this relationship statistically significant (OR = 0.50, 95% CI: 0.25, 0.99; P_trend_ = 0.049). No significant association was observed between traditional dietary patterns and the risk of depression, stress, and anxiety. There was no significant association between the western dietary pattern and the risk of depression in the un-adjusted model (OR = 1.32, 95% CI: 0.73, 2.37; P_trend_ = 0.395). After controlling for potential confounders, those in the second tertile of the western dietary pattern had higher odds for depression (OR = 2.21, 95% CI: 1.19, 4.13) compared with those in the lowest tertile; however, this association was not observed for those in the third tertile of the western dietary pattern (OR = 1.91, 95% CI: 0.95, 3.84; P_trend_ = 0.069). No significant association was observed between the western dietary patterns and risk of anxiety, either before or after adjustment for potential confounders. Higher compliance with the western dietary pattern was associated with a higher risk of stress for subjects in the second tertile compared to the lowest category (OR = 1.81, 95% CI: 1.01, 3.23), even after adjustment for sex, age, marital status, smoking, number of family members, migraine headache index score, mean arterial pressure, and physical activity (OR = 1.89, 95% CI: 1.04, 3.45). However, further adjustment for total energy intake and BMI attenuated the relationship (OR = 1.67, 95% CI: 0.90, 3.11). The association between the western dietary pattern and risk of stress was not observed in patients in the third tertile of western dietary patterns compared to the lowest category, either before or after adjustment for potential confounders.

**Table 6:** Odds ratio and 95% confidence interval for mental health (depression, anxiety and stress) according to tertiles (T) of major dietary patterns

	Healthy dietary pattern				Traditional dietary pattern				Western dietary pattern			
	T1	T2	T3	P trend	T1	T2	T3	P trend	T1	T2	T3	P trend
Depression												
Crude	Ref	0.76 (0.42, 1.36)	0.56 (0.31, 1.01)	0.107	Ref	1.10 (0.62, 1.96)	0.85 (0.47, 1.55)	0.523	Ref	1.32 (0.73, 2.37)	1.32 (0.73, 2.37)	0.395
Model 1	Ref	0.77 (0.42, 1.39)	0.55 (0.29, 1.02)	0.129	Ref	1.04 (0.58, 1.88)	0.92 (0.49, 1.78)	0.732	Ref	2.42 (1.32, 4.44) ^†^	1.91 (0.95, 3.84)	0.069
Model 2	Ref	0.72 (0.40, 1.32)	0.44 (0.22, 0.88)	0.030	Ref	0.98 (0.54, 1.79)	0.80 (0.40, 1.57)	0.563	Ref	2.21 (1.19, 4.13) ^†^	1.50 (0.69, 3.24)	0.119
Anxiety												
Crude	Ref	1.04 (0.58, 1.87)	0.99 (0.54, 1.81)	0.917	Ref	1.32 (0.73, 2.36)	1.18 (0.65, 2.14)	0.597	Ref	1.14 (0.64, 2.05)	1.00 (0.56, 1.80)	0.999
Model 1	Ref	0.95 (0.52, 1.74)	0.95 (0.51, 1.77)	0.929	Ref	1.65 (0.89, 3.05)	1.77 (0.91, 3.44)	0.098	Ref	1.16 (0.64, 2.13)	1.20 (0.60, 2.40)	0.691
Model 2	Ref	0.87 (0.47, 1.60)	0.70 (0.35, 1.39)	0.357	Ref	1.53 (0.82, 2.85)	1.47 (0.73, 2.94)	0.216	Ref	1.03 (0.55, 1.93)	0.84 (0.39, 1.82)	0.621
Stress												
Crude	Ref	0.79 (0.45, 1.40)	0.64 (0.35, 1.15)	0.222	Ref	1.26 (0.70, 2.26)	0.98 (0.55, 1.74)	0.859	Ref	1.81 (1.01, 3.23) ^†^	1.38 (0.77, 2.46)	0.292
Model 1	Ref	0.71 (0.40, 1.28)	0.62 (0.34, 1.15)	0.207	Ref	1.32 (0.72, 2.40)	1.20 (0.65, 2.22)	0.592	Ref	1.89 (1.04, 3.45) ^†^	1.56 (0.81, 3.00)	0.170
Model 2	Ref	0.69 (0.38, 1.24)	0.50 (0.25, 0.99)	0.049	Ref	1.23 (0.67, 2.25)	1.01 (0.52, 1.96)	0.862	Ref	1.67 (0.90, 3.11)	1.22 (0.59, 2.53)	0.379

Data are presented as odds ratio (95% confidence interval)

Crude: Unadjusted

Model 1: Adjusted for age, sex, marital status, number of family members, smoking status, migraine headache index score, mean arterial pressure and physical activity

Model 2: Model 2 + body mass index and energy intake per day

†*P*<0.05 was considered statistically significant

## Discussion

In the current cross-sectional study inspecting the interdependence of major dietary patterns and mental health among a sample of Iranian migraineurs, we discovered a protective association between the healthy dietary pattern with depression and stress. No significant association was observed between the traditional dietary pattern and western dietary pattern and the risk of mental disorders. Also, none of the dietary patterns showed a relationship with anxiety. This dataset is among few investigations, if not the first, exploring the associations between major dietary patterns and the mental health of migraine patients in a Middle Eastern country.

Kim and colleagues showed an inverse relationship between a healthy dietary pattern and depression risk in women after controlling for confounders [35]; however, western dietary patterns and depression were not interlinked in men and women which is consistent with our results. Another dataset in 521 municipality employees aged 21–67 years indicated less depressive symptoms in individuals on a healthy dietary pattern loaded with vegetables, mushrooms, soy products, and fruits [36]. Besides, Noguchi and colleagues found no association between western/meat dietary patterns (comprised of meat and meat products, eggs, bread, western-type cakes, biscuits, and cookies) and depression [37]. Previous reports have also confirmed that consumption of fast foods, snacks, and sugary products increases the risk of depression among the general population [38, 39].

We did not observe any association between the traditional Iranian dietary pattern and depression which is in contrast with some previous studies. A traditional Australian dietary pattern high in vegetables, fruits, beef, lamb, fish, and wholegrain lowered the possibility of depressive symptoms in adult women. However, caution is warranted when considering their finding because they failed to control the confounding effect of energy intake on depression [40]. Moreover, an inverse relationship between a traditional Norwegian dietary pattern and the risk of depression was reported in men, but not in women [21]. Also, a ‘processed food’ dietary pattern increased the risk and a ‘whole food’ dietary pattern was protective against depression in a cross-sectional sample of middle-aged British women [19]. Differences in the definition of traditional dietary patterns in each region, along with the interactions of these foods in the dietary pattern might contribute to these differences as compared to our findings.

Besides, no significant association was noted between dietary patterns and anxiety. Similar to this finding, another dataset also failed to show any connection between adherence to a Mediterranean-type diet loaded with salads, tofu, beans, yogurt, red wine, fruits, and nuts, and anxiety in 20–90 years women [41]. However, some studies counteract our results. For example, normal-weight Iranian subjects aged 20–55 years reported lower anxiety as a result of a western or traditional diet [22]. Furthermore, the odds of anxiety increased by a western dietary pattern in Norwegian adults (18). Eventually, in 50,605 middle-aged and older women, a significant direct association between adherence to the western dietary pattern and anxiety was documented [42]. Differences in study design, methods of psychological evaluation, study population, dietary assessment instruments, statistical methods, the type of covariates used, and food content of the major dietary patterns between studies could explain the discrepancies.

Despite the lack of consensus regarding the exact underlying mechanisms of the whole diet’s impact on psychological conditions, some explanations are possible. Fruits, vegetables, and whole grains are loaded with vitamins C, E, and B, folate, fiber, carotene, and diverse phytochemicals. Their roles are exerted through reduced inflammation [43], stimulated immune responses [44], and stress modulation [45], by acting as antioxidant and neural protective agents [46]. Westernized diets (full of refined grains, high fat and high sugar foods, and processed meats) increase low-grade inflammation and consequently lead to brain atrophy, which is associated with a higher chance of depression [47]. On the other hand, our traditional dietary pattern was loaded with caffeine and legumes. Legumes are rich sources of folate and other B vitamins. These nutrients might exert a positive impact on psychological conditions by lowering serum homocysteine levels as well as through the synthesis of monoamines including dopamine and serotonin in the brain [48]. When serum homocysteine rises and levels of dopamine and serotonin drop, they set the scene for increased risk of depressive disorders [48–50]. Caffeine modulates the dopaminergic pathway leading to psychostimulant effects, and adenosine receptors in the brain are acted upon by major metabolites of caffeine which may consequently result in a lower risk of depression among coffee drinkers [51]. On the other hand, our traditional dietary pattern was also characterized by a high intake of refined grains and hydrogenated fats which are associated with higher plasma concentrations of markers of inflammation [52]. Therefore, the lack of association between the traditional dietary pattern and mental health in this study might be due to the fact that these factors neutralized the protective mechanisms of caffeine and legumes.

Although this is among the first studies to examine the associations between major dietary patterns and mental health in migraine patients, some limitations need to be mentioned. First is the cross-sectional design which limits the causality. Second, participants in this study came from an urban area and resided in the same city, which has implications in terms of the sample representation and further generalization of the results. In the regression model, we controlled for some possible confounders; however, other confounders including education level and nutritional supplements were ignored. Finally, clinical diagnostic criteria are preferred to psychological screening scales used in this study to evaluate depressive symptoms and anxiety, because these scales are prone to overestimating the incidence of parameters.

## Conclusion

In summary, significant associations were documented between healthy dietary patterns and the risk of depression and stress. Current findings urge migraine patients to increase the intakes of fruits, vegetables, eggs, whole grains, nuts and seeds, meat, and poultry and reduce the intake of fast foods and snacks, processed meat, fish, cola drink, condiments, dairy, and vegetable pickles to diminish the chance of depression and stress. Further randomized clinical trials and longitudinal studies are needed to confirm the relationship between diet and mental disorders.

## Data Availability

Analysed data relevant to the study are included in the article. The datasets generated are not publicly available as set out in agreements with the commercial partners but are available from the corresponding author on reasonable request.

## Abbreviations

BMI: Body Mass Index
CI: Confidence Interval
DASS: Depression, Anxiety, and Stress Scales
FFQ: Food Frequency Questionnaire
ICHD-3: International Classification of Headache Disorders 3
IPAQ: International Physical Activity Questionnaire
OR: Odds Ratio
